# Learning from scale-up: a qualitative study of scaling-up interventions for people with multiple long-term conditions in primary care

**DOI:** 10.1093/intqhc/mzag117

**Published:** 2026-08-21

**Authors:** Sarah Chew, Natalie Armstrong, Gregory Maniatopoulos, Neil Perkins, Kamlesh Khunti, Richard Baker, Ash Routen, Janet Willars, Carolyn Tarrant

**Affiliations:** Division of Public Health and Epidemiology, George Davies Centre, University of Leicester, Leicester LE1 7RH, United Kingdom; City St George’s, University of London, London EC1V 0HB, United Kingdom; School of Business, University of Leicester, Brookfield, Leicester LE2 1RQ, United Kingdom; Division of Public Health and Epidemiology, George Davies Centre, University of Leicester, Leicester LE1 7RH, United Kingdom; Division of Global, Lifestyle and Metabolic Health, Leicester Diabetes Centre, University of Leicester, Leicester General Hospital, Gwendolen Road, Leicester, LE5 4PW, United Kingdom; Division of Public Health and Epidemiology, George Davies Centre, University of Leicester, Leicester LE1 7RH, United Kingdom; Division of Global, Lifestyle and Metabolic Health, Leicester Diabetes Centre, University of Leicester, Leicester General Hospital, Gwendolen Road, Leicester, LE5 4PW, United Kingdom; Division of Public Health and Epidemiology, George Davies Centre, University of Leicester, Leicester LE1 7RH, United Kingdom; Division of Public Health and Epidemiology, George Davies Centre, University of Leicester, Leicester LE1 7RH, United Kingdom; National Institute for Health and Care Research (NIHR) Greater Manchester Patient Safety Research Collaboration (GM PSRC), University of Manchester, Manchester M13 9PL, United Kingdom

**Keywords:** Primary Health Care, Multimorbidity, Implementation Science, Diffusion of Innovation, Qualitative Research

## Abstract

**Background:**

Scaling innovations for people living with multiple long-term conditions (MLTCs or multimorbidity) remains challenging, particularly in primary care, where organisational capacity, infrastructure, and local contexts vary widely. There is limited empirical understanding of the implementation work needed to navigate these contextual influences when scaling complex innovations across diverse settings. This study aimed to identify and explain the contextual conditions and adaptive implementation strategies that enabled or constrained scale-up of interventions for people with MLTCs in English primary care.

**Methods:**

We conducted a theory-informed qualitative evaluation of four national projects that aimed to scale evidence-based interventions to improve the care of people with MLTCs in primary care. Projects ran between 2021 and 2024. Data included longitudinal interviews with project teams, document analysis, and a stakeholder workshop. Analysis was informed by the Consolidated Framework for Implementation Research.

**Results:**

Four key themes were identified: (1) scaling requires relational infrastructure: relationships, trust, and relational continuity were central to gaining traction; (2) supporting organizations and networks facilitate spread; (3) engaging primary care practices depends on addressing uneven readiness for research and innovation; and (4) local intelligence supports implementation at scale.

**Conclusion:**

Scaling MLTC innovations in primary care is shaped by relational, organisational, and infrastructural factors, including uneven readiness for implementation. Scaling proven interventions requires technical support and investment in the people, relationships, and local infrastructures that support embedding across diverse primary care contexts.

## Introduction

The challenges of spreading, scaling up, and sustaining healthcare improvements are well documented [1, 2], reflecting the difficulty of translating successful interventions into broader clinical practice. The World Health Organization defines scaling up as ‘deliberate efforts to increase the impact of health service innovations successfully tested in pilot or experimental projects so as to benefit more people and to foster policy and programme development on a lasting basis’ [3]. This highlights that scaling requires proactive planning and careful adaptation to diverse contexts, with ongoing management of local system and workforce factors. Implementation science frameworks, including the Consolidated Framework for Implementation Research (CFIR) [4] and Promoting Action on Research Implementation in Health Services (PARiHS) [5], highlight the need to consider context in planning for and evaluating implementation.

The Dynamic Sustainability Framework argues that sustainability depends on ‘continued learning and problem solving, [and] ongoing adaptation of interventions with a primary focus on fit between interventions and multi-level contexts’ [6]. Implementa­tion science increasingly recognises the complex, relational, contingent, and situated nature of implementation [7, 8]. Despite these developments, there remains limited empirical insight into how such complexity is navigated when implementing interventions at scale, particularly in primary care [9, 10]. Primary care is characterised by its generalist nature, practice autonomy, pressures and incentives, and diverse working relationships, all of which can conflict with research and innovation goals [11].

Primary care in England is operating under intense and uneven pressure. General practices vary widely in organisational form, workforce capacity, staffing models, and digital infrastructure, resulting in substantial differences in readiness for implementation. Recent system reforms, including the Health and Care Act 2022 and the establishment of Integrated Care Boards (ICBs), have been accompanied by significant reductions in system-level capacity [12]. NHS England announcements in 2023 and 2025 mandated major reductions in ICB running costs, leading to workforce losses and reconfiguration of commissioning functions through clustering and mergers [13]. While intended to support integration and efficiency, these reforms have also led to organisational instability and reduced local capacity, further complicating efforts to implement and scale innovations in primary care.

At the same time, the rising prevalence of patients with multiple long-term conditions (MLTCs, also known as multimorbidity) poses a growing challenge for primary care [14, 15]. People with MLTCs are highly diverse, often with complex clinical and social needs that do not fit neatly within single-condition models of care, and are commonly supported through multidisciplinary working. Designing and scaling interventions that meet these challenges requires attention not only to intervention content, but also to the social and political work of embedding them in context.

This study uses English primary care as an analytically informative case through which to examine how contextual conditions and adaptive implementation strategies shape the scaling up of complex interventions for people living with multiple long-term conditions in heterogeneous primary care settings. In doing so, it focuses on the often-invisible implementation work required to translate interventions across diverse organisational and practice contexts.

Drawing on four nationally funded programmes that aimed to implement at scale a range of interventions to improve care for people with MLTCs in primary care in England, the aim was to identify and explain the contextual conditions and adaptive implementation strategies that enabled or constrained scale-up.

## Materials and methods

We conducted a longitudinal qualitative evaluation, using semi-structured interviews and thematic analysis, of four large-scale research projects seeking to implement at scale interventions designed for patients with MLTCs in English primary care. The study was informed by an interpretivist approach and guided by CFIR as a sensitising framework.

**Figure 1:**
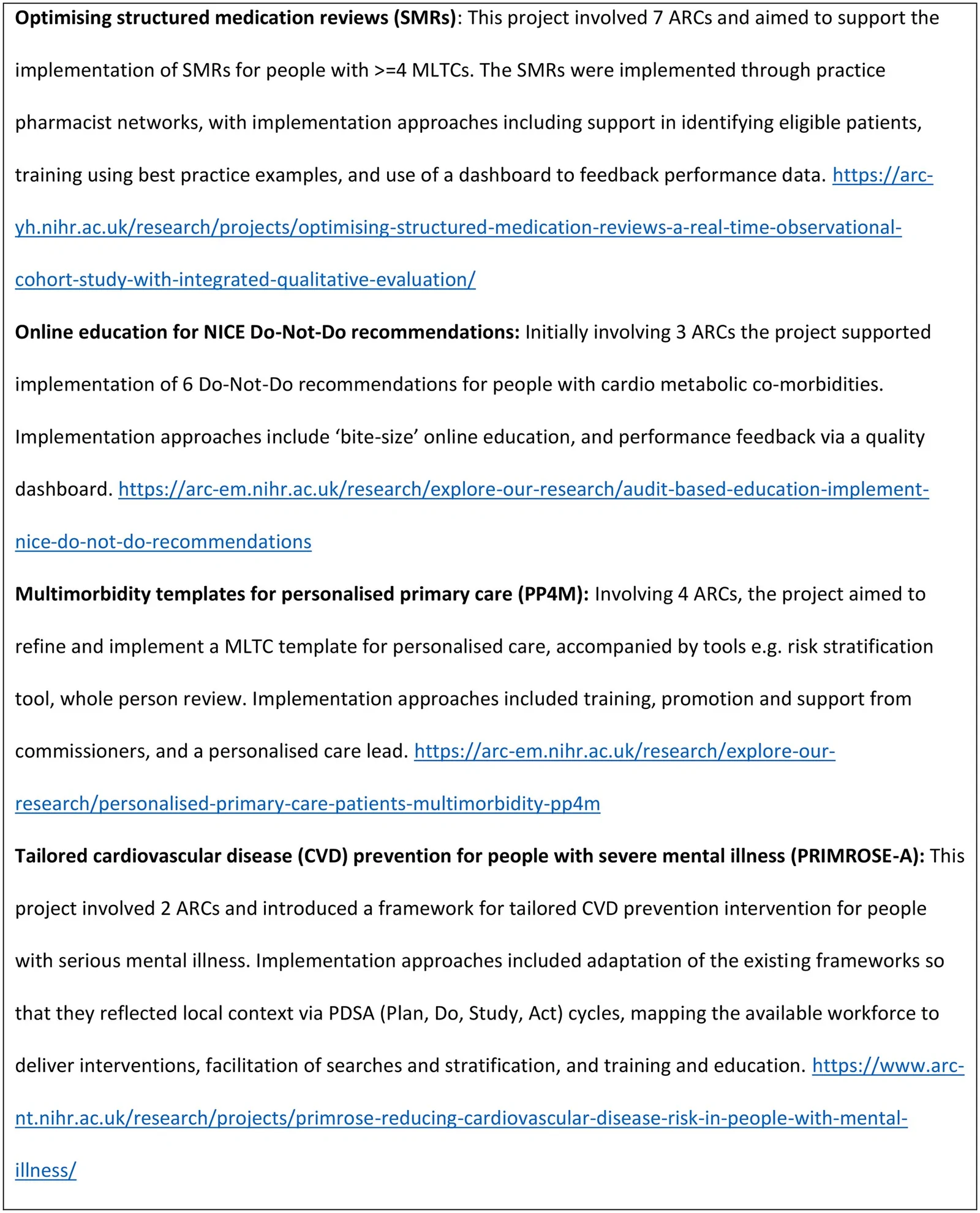
Overview of the four scaling up projects undertaken across England between 2021 and 2024.

Each of the four implementation projects was supported through a national programme of funding between 2021 and 2024. The interventions were aligned with global/national policy responses to MLTCs, designed to improve aspects of MLTC care, and had previous evidence of effectiveness. Each project was led from one of the regional National Institute for Health and Care Research Applied Research Collaborations (ARCs), which support applied health and care research responsive to local populations and systems, and involved implementation across several ARC regions (see Fig. 1). Core teams within the lead ARCs led practice recruitment, local implementation support, and implementation evaluation. The project teams included experienced implementation scientists and had support via implementation and academic networks. The qualitative study was undertaken by nonclinical, social science researchers experienced in qualitative research and implementation science, with advice from two academic GPs with interest in MLTCs. The qualitative study team participated in cross-project meetings but, otherwise, were independent from the implementation project teams.

Sampling was limited to members of the four project teams. Participants were recruited by contacting each project lead and asking them to identify individuals best able to provide insight into implementation at scale; additional participants were identified through snowball sampling. Interview participants were selected from core team members to include project leads, researchers, and implementation experts. A topic guide was used and revised iteratively (Supplementary Appendix A). Initial interviews explored project focus, intervention features, implementation strategies, and stakeholder relationships. Subsequent interviews explored experiences of implementation at scale, how strategies evolved, barriers, enablers, and experiences of success. Interview rounds were undertaken in Autumn 2022, Spring 2023, and Spring 2024. Interviews were conducted by nonclinical researchers (S.C., J.W.) over Microsoft Teams or by telephone, audio-recorded and transcribed verbatim. Documents included implementation plans, meeting notes, and project presentations and reports.

We took a theory-informed thematic approach to analysis [16], using the inner and outer context domains of CFIR as sensitising concepts to support initial coding and interpretation. This structured our exploration of how local conditions shaped scale-up efforts while retaining openness to emergent themes. Inner context domains included structural characteristics, networks and communication, implementation climate, readiness for implementation, and access to information. Outer context domains included external policy and incentives, patient needs and resources, and cosmopolitanism. Coding was undertaken by S.C., with theme development by S.C. and C.T. (nonclinical social scientists). NVivo supported initial open coding, then coded data quotes were extracted and organized into themes using mind maps and theme summaries, drawing on CFIR. Case summaries were produced for each project, informed by interview analysis and document review, and updated after each interview round to account for temporal change. Data were synthesized and interpreted across cases using thematic narrative summaries.

Emerging findings were sense-checked through presentations to cross-project team meetings during the study. A final workshop was held (August 2024) involving six project stakeholders to test and refine findings, enhancing the trustworthiness of the findings.

### Ethical issues

This study was approved by the University of Leicester Medicine and Biological Sciences Research Ethics Committee (Ref: 32308-sec55-ls:healthsciences). Informed consent was secured from all participants, and interview recordings were anonymized to remove names or identifying details of individuals or organisations prior to analysis.

## Results

Nineteen interviews were completed across the four projects, with between three and six interviews per project. Three were paired interviews. Interviews included 13 unique individuals: 3 were interviewed three times, 3 twice, and 7 once. Participants included two clinical project leads, five health services research fellows or research facilitators, and six implementation fellows or implementation researchers (Table 1).

**Table 1:** Interview participants.

Participant identification	Project	No. of times interviewed
P1	NICE Do-Not-Do, SMRs	3
P2	NICE Do-Not-Do	3
P3	PRIMROSE-A	3
P4	PP4M	2
P5	SMRs	2
P6	PP4M	2
P7	PRIMROSE-A	1
P8	SMRs	1
P9	PP4M	1
P10	PP4M	1
P11	NICE Do-Not-Do, SMRs	1
P12	PRIMROSE-A	1
P13	PRIMROSE-A	1

Four key themes related to implementation work for scale-up within the primary care context were identified: (1) scaling requires relational infrastructure; (2) working with supporting organisations and networks facilitates implementation at scale; (3) engaging primary care practices depends on addressing uneven readiness for research and innovation; and (4) scaling requires leveraging local intelligence. Mapping of themes to CFIR constructs is provided in Supplementary Appendix B. Illustrative quotes supporting each theme are provided in Table 2.

**Table 2:** Data supporting scaling-up interventions for people with multiple long-term conditions in the primary care.

Theme	Representative quotations
Scaling requires relational Infrastructure	Q1: I think with engagement with GP practices, specifically having champions and people who really believed in the intervention and were there, you know, they were willing to give time and effort to support it and drive it locally. I think that played a major role in engagement as well, like facilitating it. (P12)
	Q2: You have to have somebody whose language can boundary span. To win them over because sending them a link to see a GP talking about long term condition reviews is never going to win over nurses. (P4)
	Q3: It’s a challenge, you know, because champions move on, the champions get other things [put] onto their portfolios. (P13)
	Q4: I don’t think we’ve really expected so much iteration and change. So for example, with stakeholder mapping, I don’t think we expected to. I mean, we could have done a different stakeholder map every month. (P3)
Working with supporting organizations and networks facilitates spread	
	Q5: I think we’ve been slightly lucky that we managed to reach out quite early on. To [the clinical lead for topic] for the entire CCG in [location]; she is also the clinical director of one of the primary care networks. […] So engaging with her was key and that happened very early on. […] She’s actually provided a lot of the connections. (P3)
	Q6: So people within the [condition] team within the Academic Health Science network [have been critical]. In terms of doing the initial engagement piece, the initial ‘Ok how does this work within your area?’, that mapping and then a little bit more in terms of direct communication with the individual practices who are implementing. […] Part of the reason [this happened is because] I have one foot very much within the implementation, because I work partly [in Academic Health Science network] as well as partly within the ARC (P7)
	Q7: It isn’t just you trying to engage a CCG now. There’s lots of different areas, isn’t there? And it’s hard to pin down. (P4)
	Q8: In [location] [Organisation] is one of the largest federations of groups of practices bigger than a primary care network. It covers most of the practices in and around [location]. […] and that’s increasingly becoming the level at which innovations happen and they developed them, roll them out. All the partner practices and we’ve done some research projects [with them] and that could be because that’s a big enough organisation who are interested in. […] But that data collection and the management for sort of management purposes you know, so they set up systems and sharing access to records and be able to roll out. […] We’ve had a productive relationship with them (P6)
	Q9: I think we’ve seen already some early evidence of kind of spread and for example with our project actually we started with two [local locations] and then a site up in [non-local location] and actually organically through kind of word of mouth. (P7)
Engaging primary care practices depends on addressing uneven readiness for research and innovation	
	Q10: I think that the difference is that the people who joined [Project] are already within the network. So they’re part of the [research] centre already. And usually, the types of practices that sign up to studies like this are quite experienced practices who have taken part in different studies in the past and so on […] Yeah, they’re definitely more research ready. […] And so what we’ll find is that they’re quite set up already for studies and they use them as revenue. (P2)
	Q11: Usually the types of practices that sign up to studies like this are quite experienced practices who have taken part in in different studies in the past and so on. […] quite sort of study veterans (P3)
	Q12: Part of our sell is […] I showed that these people often will have like […] ten different appointments anyway through the year, review appointments because of calling them up for separate diseases. So, [although the intervention involves] calling them for one or two longer appointments [this] isn’t necessarily gonna take longer than ten short appointments and we showed how for typical patients that would be true. Ok you’re doing things you weren’t doing before but you’re not wasting lots of time asking about smoking ten times a year. So there are efficiency gains and what we showed […] is that it didn’t actually cost anymore, based on peoples’ time, than conventional ways of working (P6)
	Q13: The focus of the study is how can we help practices actually roll this out and do it and what stops us… what stops practices doing it what makes it easier to do it and so on. (P1)
	Q14: One nurse…said to us we’ve been wanting to do this for ages with long term conditions. We’ve been saying we wanted to do this for ages and haven’t known how to do it because we’re seeing patients in isolation. We’re repeating the same message often for several conditions and we’ve kept saying if only we’d got a multimorbidity template or we could merge a lot of this. So, when we showed them that they’d had this template on the screen. All this time it was on their system they were kicking themselves you know I can’t believe that that’s been there all along (P9)
Leveraging local intelligence supports implementation at scale	
	Q15: A lot of the training videos and a lot of the recruitment videos […] utilised GPs to deliver the training videos and the photographs were of GPs. When actually I knew the people that were going to be using this template the most were the nurses. (P4)
	Q16: We haven’t really specified [who will lead on the ground] because every practice has got different arrangements. It depends on the availability. Some have got their own pharmacists, some share pharmacists, some haven’t got a pharmacist at all. (P6)
	Q17: With that, they also changed the way that they provide data, which meant that we had to make a lot of changes our ends in terms of how we receive the data and what we do with it. But basically, it meant the practices we initially thought initially extracting data reliably from, we were no longer receiving data reliably from some of them. We then had to do investigative work to find out which ones we were receiving data for and then [person] had to recruit some additional practices so that took time. (P1)
	Q18: So we’ve got two […] sites who are probably going to have slightly different workforce available to implement different elements. (P6)

### Scaling requires relational infrastructure

Scaling emerged less as a process of replication and more as a process of negotiation, relationship building and adaptation. Building trust, aligning with local values, working closely with local champions, and repeatedly re-engaging stakeholders was essential for successful implementation.

Working with locally embedded champions has long been recognized as key to gaining traction in implementation [17]. Across the four innovation projects, successful scale-up relied on identifying and engaging individuals who acted as relational anchors—local champions drawn from local primary care practices and the wider health system, who invested time, credibility, and energy in mobilising stakeholders, convening meetings, and sustaining engagement. Champions facilitated alignment across disciplines, bridged structural divides and acted as ‘boundary spanners’ [18], facilitating transactions and information between individuals, groups and organisations. Effective champions also acted as advocates and system navigators, drawing on deep knowledge of local structures to support integration of interventions (Q1, Q2).

However, such relational infrastructure proved fragile. Local champion roles were often informal and did not come with dedicated funding or protected time. Structural reforms and workforce turnover made it particularly difficult for project teams to establish and retain local champions for the duration of their projects. Staff turnover frequently resulted in the sudden loss of key champions, undermining continuity and momentum (Q3).

Engagement with stakeholders within and beyond practices was needed, but efforts to map and engage stakeholders were challenged by the unexpected and unprecedented scale of ongoing changes in the system (Q4).

Maintaining relational infrastructure in the face of this turbulence required extensive additional work from project teams. Often teams were not aware of personnel changes until they contacted a site. They then had to identify and build relationships with new personnel, bringing them up to speed and building their engagement and motivation to support the project.

### Working with supporting organisations and networks facilitates spread

Where teams could draw on support from commissioning and planning bodies, formerly Clinical Commissioning Groups (CCGs) and latterly ICBs, and national improvement networks such as Academic Health Science Networks (now Health Improvement Networks), this strongly facilitated implementation at scale, including cross-region implementation. Proactive engagement with these organisations, and the use of agents able to connect across organisations, were perceived as key facilitators (Q5, Q6).

Implementation team members, however, highlighted how it was increasingly difficult to identify decision-makers or sustain engagement due to evolving national infrastructure and staff turnover. Restructuring disrupted established commissioning relationships and fragmented infrastructure that many projects had relied on to support spread. This required additional work to understand changes to the remit and responsibilities of restructured organisations and individuals, identify which organisations and staff were best placed to support implementation at scale, and build new lines of engagement with newly formed organisations (Q7).

Existing Primary Care Networks, formal NHS groupings of GP practices working together to deliver coordinated primary care services, and local research interest networks were valuable for supporting implementation at scale. Established relationships and reputation facilitated engagement, and there were examples of spread happening organically through word of mouth. When teams worked outside their region, personal contacts and access to external Primary Care Networks were similarly facilitative (Q8, Q9).

### Engaging primary care practices depends on addressing uneven readiness for research and innovation

One challenge for implementation teams was extending the reach of their implementation efforts to practices that were less ready for research and innovation. While some practices had strong links with research-active institutions and were embedded in research networks, others were more isolated. Well-networked and resourced practices with prior experience in research and innovation were more likely to engage. In contrast, less research-ready practices had limited experience, placed lower priority on innovation, and reported insufficient time or resources to participate in improvement activities beyond routine service delivery. Without targeted support, these sites risked being left behind in national innovation efforts. Teams noted engaged practices were often the ‘frequent flyers’ of research and innovation (Q10, Q11).

For practices less ready for research and innovation, strategies included identifying local champions within a practice or at the CCG/ICB or Primary Care Network level to support engagement. While financial incentives were expected to help, they were often ineffective compared with hands-on support. More effective strategies involved demonstrating how an intervention could improve efficiency or save time, alongside practical support for implementation. These approaches addressed real-world pressures and helped overcome barriers related to limited experience and capacity (Q12, Q13).

There were also efforts to ‘sell’ innovations to practices by showing how they could be achieved with little or no additional resource; for example, one respondent cited a practice that did not realise a template for recording MLTC was already on its computer system. Question and answer events, aligning with practices’ objectives, and identifying a niche gap of interest to practices were other strategies identified (Q14).

### Leveraging local intelligence supports implementation at scale

Scaling in primary care is not simply a technical or procedural endeavour. It is a practice of translation—requiring knowledge of local infrastructures including digital architecture, service flows, organisational histories, and informal workarounds. Such knowledge is typically tacit, situated, and difficult to capture in centralised guidance or logic models. Teams needed to draw on intelligence about local services and to revisit and adapt either the interventions themselves and/or the ways they were implemented (Q15, Q16).

The teams’ connection to local intelligence was critical for identifying changes, such as internal restructuring or changes to IT infrastructure, that affected implementation. Several participants described IT system providers changing data formats, extraction methods, or access protocols, disrupting established workflows. One example was a supplier rebrand that modified data-sharing infrastructure, leading to sudden loss of reliable data extraction from some practices and requiring additional work to identify affected sites and, in some cases, recruit alternatives (Q17).

Across the projects, success was often tied to individuals or small teams within primary care networks or practices who had previous implementation experience and deep local intelligence about ‘how things actually work here’. This included awareness of the workforce, system boundaries, referral pathways, digital architecture, local data quirks, and gatekeeping arrangements—knowledge crucial for tailoring interventions while preserving fidelity of function (Q18).

Unlike the broader relational work discussed earlier, this form of local knowledge was often practical and procedural, concerned with how systems, routines, data flows, and everyday work were organised in particular places. Some implementers drew on their understanding of local practice workflows to adapt digital templates; others identified when a site appeared on board but had a history of nonengagement. These insights made scale-up more feasible because they allowed adaptation in real time.

There were, however, limitations to relying on this form of intelligence when implementing at scale. It was often embodied in a single individual and could be lost through role changes, practice closures, or system change. Unlike formal implementation strategies, these fragments of insight were not written down, budgeted for, or transferred between teams, and their loss led to duplicated effort and slower progress. This loss of ‘organisational’ or ‘corporate’ memory is an often-cited reason for the faltering or breaking down of collaborative working [19], with key individuals either being relocated or exiting the health arena, resulting in loss of expertise as well as local champions.

## Discussion

### Statement of principal findings

This study used CFIR’s inner and outer context domains to explore the complexities of scaling innovations in primary care, showing that scaling is not simple replication but a dynamic, socially embedded, and contextually sensitive endeavour. Themes mapped primarily onto CFIR constructs related to partnerships, relational connections, and communications. Successful scale-up required sustained relational work: building and maintaining relationships, supporting local champions, utilising supporting organizations and primary care networks, and acting on local knowledge, while navigating uneven readiness for implementation. This was shaped by resource constraints, variable digital architecture, and structural reforms.

### Strengths and limitations

Strengths of this study are the use of qualitative methods to uncover mechanisms behind the adaptive and iterative process of scaling up [20]. We included four distinct projects within a national programme and used a theory-based approach to studying implementation at scale. We were only able to explore the experiences of implementation teams: due to exceptional pressures on primary care during the post-COVID environment and the ongoing service reconfiguration prompted by the 2022 Health and Social Care Act (Department of Health and Social Care, 2022), we were unable to interview healthcare professional participants in each of the four projects as originally planned. Our sampling approach was constrained by the focus on the four specific project teams and snowball sampling, and findings are context-specific. All four projects were ARC-led and supported by substantial implementation expertise and infrastructure. This infrastructure is itself part of the mechanism for successful scale-up, and findings may be less transferable to situations where such support is limited or absent.

Nevertheless, while grounded in English primary care, the study identifies implementation mechanisms—relational infrastructure, organizational readiness, and the use of local intelligence—that are likely to be relevant to other health systems with heterogeneous primary care capacity. The findings therefore offer transferable insights into the conditions required to scale complex interventions for people living with multiple long-term conditions across diverse primary care contexts.

### Interpretation within the context of wider literature

The importance of relational infrastructure for implementation at scale has been recognized by others: Koorts *et al.* in their realist review of mechanisms for successful scaling up, identified relationships with local and national stakeholders as integral to scale-up success, with community sustainability/embeddedness and stakeholder buy-in/perceived value making significant contributions to successful scale-up [21]. Equally critical is the role of tacit local knowledge held by key individuals who understand workflows, workforce configurations, and informal system adaptations, enabling tailoring of interventions while preserving core functions. However, ongoing re-organisations and restructuring in the NHS risk destabilising relationships and partnership working more generally [22]. Furthermore, the knowledge required to adapt and translate interventions and implementation strategies is often fragile and poorly institutionalised, making scaling efforts vulnerable to disruption amid workforce turnover and system change.

Scaling innovation requires moving beyond early adopters to engage practices facing greater capacity and contextual challenges, yet this was not always straightforward. Our study highlights the need for diverse engagement strategies, rather than reliance on existing research networks, and for recognising what motivates practices to participate. For less research-ready practices, financial incentives alone had limited traction; engagement depended on reframing innovations in terms of efficiency and time savings, alongside practical, hands-on implementation support. External facilitation and webs of support have been identified as critical for reaching hard-to-engage sites [23]. This often-invisible implementation work was enabled here by funding but may be difficult to sustain in unfunded scale-up efforts.

Our findings align with primary care implementation research showing that competing priorities, limited capacity, and fit with everyday practice shape engagement with improvement initiatives [11]. A systematic review of chronic care model implementation in primary care [24] similarly highlights networks and organisational readiness as supporting implementation, issues reinforced by studies of guideline implementation in primary care [25]. Our study extends this work by examining how these dynamics operate during wider scale-up across multiple interventions, highlighting the relational and adaptive work required to engage diverse practices.

Together, our findings underscore that scaling innovations demand negotiation, adaptation, and investment not only in technical processes but also in people, relationships, and local implementation support—balancing hard and soft assets [2]. They reinforce scholarship that conceptualises scaling as a complex, adaptive process embedded in social and organizational contexts [26, 27] and align with research highlighting the importance of social capital for scaling up [28]. Similar to critiques of staged or linear models [29], our results foreground relational and adaptive work often relegated to the margins of implementation frameworks.

### Implications for policy, practice, and research

Scaling up requires long-term investment and sustained policy commitment, yet continuous organizational change, workforce pressures, and resource scarcity make this challenging. As Chamberlain *et al.* noted: ‘We now know that implementation of new programs into usual services can sometimes take years and require changes at multiple levels of the system. Those changes must be integrated into local commissioning and delivery structures and cannot merely reside in the goodwill and efforts of champions for the programs’ [30]. At a national level, dedicated and accessible infrastructure is essential to facilitate spread in primary care. Policymakers and funders should resource relational work, including trust-building, alignment, and ongoing engagement, and ensure hands-on implementation support for hard-to-engage practices. This work should be planned, funded, and evaluated as part of scale-up, rather than treated as informal or discretionary effort. Within restructuring, attention is needed to how local contextual knowledge can be preserved, curated, and transferred. Scaling complex interventions requires negotiation, tailoring, and persistence—work that is essential but often invisible. Recognizing, valuing, and resourcing this work is critical to ensuring innovations are adopted, adapted and sustained across diverse settings.

## Conclusions

Efforts to scale innovation in primary care must attend to the dynamic and contingent nature of the social, organizational, and infrastructural conditions in which they are implemented. Our findings suggest that scale is not a straightforward process of applying implementation strategies across a wider range of settings but a process of social embedding, adaptation, and navigation of inner and outer system dynamics. They highlight the importance of relational infrastructure, organizational networks, navigation of uneven readiness and contextual constraints, and access to local intelligence. Scaling proven interventions requires technical support and sustained investment in the people, relationships, and local infrastructures that enable them to take root, endure, and deliver improvement across diverse primary care settings.

## Supplementary Material

mzag117_Supplementary_Data

## Data Availability

The datasets generated and/or analysed during the current study contain information that could identify participants. To protect confidentiality, these data are not publicly available.

## Data Availability

The data underlying this article are not publicly available due to the sensitive and potentially identifiable nature of the qualitative data. De-identified data may be available from the corresponding author on reasonable request, subject to ethical approval and appropriate data-sharing agreements.

## References

[1] Greenhalgh T , Robert G, Macfarlane F et al Diffusion of innovations in service organizations: systematic review and recommendations. Milbank Q 2004;82:581–629. 10.1111/j.0887-378X.2004.00325.x 15595944 PMC2690184

[2] Côté-Boileau É , Denis J-L, Callery B et al The unpredictable journeys of spreading, sustaining and scaling healthcare innovations: a scoping review. Health Res Policy Syst. 2019;17:84. 10.1186/s12961-019-0482-6 31519185 PMC6744644

[3] World Health Organization. Practical Guidance for Scaling Up Health Service Innovations. Geneva: World Health Organisaiton; 2009.

[4] Damschroder LJ , Reardon CM, Widerquist MAO et al The updated consolidated framework for implementation research based on user feedback. Implement Sci. 2022;17:75. 10.1186/s13012-022-01245-0 36309746 PMC9617234

[5] Kitson A , Harvey G, McCormack B. Enabling the implementation of evidence based practice: a conceptual framework. Qual Health Care 1998;7:149–58. 10.1136/qshc.7.3.149 10185141 PMC2483604

[6] Chambers DA , Glasgow RE, Stange KC. The dynamic sustainability framework: addressing the paradox of sustainment amid ongoing change. Implement Sci 2013;8:117. 10.1186/1748-5908-8-117 24088228 PMC3852739

[7] Greenhalgh T , Papoutsi C. Spreading and scaling up innovation and improvement. BMJ. 2019;365:l2068. 10.1136/bmj.l2068 31076440 PMC6519511

[8] Lanham HJ , Leykum LK, Taylor BS et al How complexity science can inform scale-up and spread in health care: understanding the role of self-organization in variation across local contexts. Soc Sci Med 2013;93:194–202. 10.1016/j.socscimed.2012.05.040 22819737

[9] Ben Charif A , Zomahoun HTV, LeBlanc A et al Effective strategies for scaling up evidence-based practices in primary care: a systematic review. Implement Sci 2017;12:139. 10.1186/s13012-017-0672-y 29166911 PMC5700621

[10] Corôa RDEC , Gogovor A, Ben Charif A et al Evidence on scaling in health and social care: an umbrella review. Milbank Q. 2023;101:881–921. 10.1111/1468-0009.12649 37186312 PMC10509507

[11] Armstrong N , Herbert G, Brewster L. Contextual barriers to implementation in primary care: an ethnographic study of a programme to improve chronic kidney disease care. Fam Pract 2016;33:426–31. 10.1093/fampra/cmw049 27297465 PMC4957013

[12] Maniatopoulos GH , Hunter DJ. The health system in the United Kingdom: past, present and future. In: International Encyclopedia of Public Health. 3rd ed. London: Elsevier, 2025, 683–92. 10.1016/B978-0-323-99967-0.00195-2

[13] National Confederation of Health and Social Care . *ICB Clusters and Mergers: What You Need to Know* [Internet]. London: NHS Confederation; 2025 [cited 2026 Aug 28]. https://www.nhsconfed.org/publications/icb-clusters-and-mergers

[14] Whitty CJ , MacEwen C, Goddard A et al Rising to the challenge of multimorbidity. BMJ. 2020;368:l6964. 10.1136/bmj.l6964 31907164 PMC7190283

[15] Bisquera A , Turner EB, Ledwaba-Chapman L et al Inequalities in developing multimorbidity over time: a population-based cohort study from an urban, multi-ethnic borough in the United Kingdom. Lancet Reg Health Eur 2022;12:100273. 10.1016/j.lanepe.2022.100273 34901910 PMC8640725

[16] Braun V , Clarke V. Reflecting on reflexive thematic analysis. Qual Res Sport Exerc Health 2019;11:589–97. 10.1080/2159676X.2019.1628806

[17] Pettersen S , Eide H, Berg A. The role of champions in the implementation of technology in healthcare services: a systematic mixed studies review. BMC Health Serv Res 2024;24:456. 10.1186/s12913-024-10867-7 38605304 PMC11007964

[18] Haas A. Crowding at the frontier: boundary spanners, gatekeepers and knowledge brokers. J Knowl Manag 2015;19:1029–47. 10.1108/JKM-06-2015-0243

[19] Hunter DJ , Perkins N, Bambra C et al Partnership Working and the Implications for Governance: issues Affecting Public Health Partnerships. London: NIHR Service Delivery and Organisation Programme, 2011.

[20] Van Grootven B , Sibilio S, Curreri N et al Methodological considerations for evaluating scale-up programmes in healthcare: a methods review. Int J Qual Health Care 2026;38:mzaf120. 10.1093/intqhc/mzaf120 41358939 PMC12790437

[21] Koorts H , Cassar S, Salmon J et al Mechanisms of scaling up: combining a realist perspective and systems analysis to understand successfully scaled interventions. Int J Behav Nutr Phys Act 2021;18:42. 10.1186/s12966-021-01103-0 33752681 PMC7986035

[22] Perkins N , Hunter DJ. Health and wellbeing boards: a new dawn for public health partnerships? J Integr Care 2014;22:220–9. 10.1108/JICA-07-2014-0030

[23] Miake-Lye I , Mak S, Lam CA et al Scaling beyond early adopters: a content analysis of literature and key informant perspectives. J Gen Intern Med 2021;36:383–95. 10.1007/s11606-020-06142-0 33111242 PMC7878615

[24] Kadu MK , Stolee P. Facilitators and barriers of implementing the chronic care model in primary care: a systematic review. BMC Fam Pract 2015;16:12. 10.1186/s12875-014-0219-0 25655401 PMC4340610

[25] Wang T , Tan J-YB, Liu X-L et al Barriers and enablers to implementing clinical practice guidelines in primary care: an overview of systematic reviews. BMJ Open 2023;13:e062158. 10.1136/bmjopen-2022-062158 PMC9827241 36609329

[26] May CR , Johnson M, Finch T. Implementation, context and complexity. Implement Sci 2016;11:141. 10.1186/s13012-016-0506-3 27756414 PMC5069794

[27] Maniatopoulos G , Hunter DJ, Erskine J et al Large-scale health system transformation in the United Kingdom: implementing the new care models in the NHS. J Health Organ Manag 2020;34:325–44. 10.1108/JHOM-05-2019-0144 PMC7406990 32181995

[28] Rotteau L , Albert M, Bhattacharyya O et al Understanding decisions to scale up: a qualitative case study of three health service intervention evaluations. J Health Serv Res Policy. 2021;26:37–45. 10.1177/1355819620921892 32380915

[29] Niang M , Alami H, Gagnon M-P et al A conceptualisation of scale-up and sustainability of social innovations in global health: a narrative review and integrative framework for action. Glob Health Action. 2023;16:2230813. 10.1080/16549716.2023.2230813 37459240 PMC10353334

[30] Chamberlain P , Roberts R, Jones H et al Three collaborative models for scaling up evidence-based practices. Adm Policy Ment Health 2012;39:278–90. 10.1007/s10488-011-0349-9 21484449 PMC4312010

