# Supplementary material for: Learning from scale-up: a qualitative study of scaling-up interventions for people with multiple long-term conditions in primary care
Source: Int J Qual Health Care. 2026 Aug 21;38(3):mzag117. doi: 10.1093/intqhc/mzag117 (PMC13552373; doi:10.1093/intqhc/mzag117)
Supplement: Supplementary file 1 [file mzag117_supplementary_data.zip › Supplementary material Appendix A.docx]

Supplementary material: Appendix A

**Topic Guide for Interviews**

**1: The participant**

- What is your role in the project?
- How would you like us to refer to you in our outputs?

**2.The project & intervention**

- Can you give me a brief overview of how the project came about?
- Can you describe the intervention and intended outcomes, what is the driver for this intervention?
- To what extent has this intervention already been implemented? What has been learned from this?

**3.Implementation**

- Can you tell me what taking part in the project has involved for you?
- Can you tell me about the implementation plan? What strategies are being used to encourage or support implementation?
- How has implementation been going so far? What has worked well/less well, and why?
- Is there anything that has been particularly important in supporting local implementation?
- What, if any, difficulties have arisen with implementing the intervention? How are these problems being addressed? (probe for features of the intervention, and barriers to implementation)
- How do people feel about the intervention? Has the intervention had to be adapted in any way? How and why?
- Is there variation in engagement and uptake across the organisations involved? Why? (OR, depending on role - Is there anything specific about the context you are working in that impacts on engagement and uptake?)
- Who do you think are the *key stakeholders* in making implementation happen? Do you feel they are all on board? Why?
- What *funding* is needed and how is this being arranged? How does this impact on implementation?
- What do you feel is important for *sustainability?*
- What is your experience of being involved in implementation at scale as part of a large group of organisations, does this bring any benefits?
  - To what extent do you feel part of a community of people involved in implementing the intervention? What difference (if any) does this make?
- How easy is it for you to get advice and support in addressing implementation challenges when they arise? Where do you get this from?
- How have you found the support from the project team? What role has this played in the implementation process?
- Thinking about the project as a whole, is there anything about the approach being taken to roll out the intervention that you would say is particularly useful or effective?
- Is there anything that could be improved?

**4: Closing question**

Is there anything that I haven’t asked about that you feel would be useful for us to know?
