# Supplementary material for: Learning from scale-up: a qualitative study of scaling-up interventions for people with multiple long-term conditions in primary care
Source: Int J Qual Health Care. 2026 Aug 21;38(3):mzag117. doi: 10.1093/intqhc/mzag117 (PMC13552373; doi:10.1093/intqhc/mzag117)
Supplement: Supplementary file 1 [file mzag117_supplementary_data.zip › Supplementary material Appendix B 270726.docx]

**Appendix B: Mapping themes to CFIR concepts**

| Study theme | Best-fit CFIR construct |
| --- | --- |
| Scaling requires relational infrastructure | Relational Connections; Communications; Work Infrastructure (Inner Setting) |
| Working with supporting organisations and networks | Partnerships and Connections (Outer Setting) |
| Engaging practices depends on uneven readiness | Partnerships and Connections (Outer Setting); Available Resources (Inner Setting) |
| Leveraging local intelligence | Relational Connections; Communications (Inner Setting) |
